# Author Correction: A split fluorescent reporter with rapid and reversible complementation

**DOI:** 10.1038/s41467-019-11689-6

**Published:** 2019-08-14

**Authors:** Alison G. Tebo, Arnaud Gautier

**Affiliations:** PASTEUR, Department of Chemistry, École Normale Supérieure, PSL University, Sorbonne University, CNRS, 75005 Paris, France

**Keywords:** Assay systems, Proteins

Correction to: *Nature Communications* 10.1038/s41467-019-10855-0, published online 27 June 2019.

The authors became aware of a mistake in the original version of this Article. Specifically, the fusion proteins ‘FRB-NFAST’ and ‘FKBP-CFAST*n*’ were incorrectly referred to as ‘FKBP-NFAST’ and ‘FRB-CFAST*n*’, respectively, in a number of places. As a result of this, the following changes have been made to the originally published version of this Article:

In Fig. 1b and Fig. 1c, the vertical labels on the right-hand side originally incorrectly read ‘FKBP-N + FRB-C’ instead of the correct ‘FRB-N + FKBP-C’.

The third sentence of the caption to Fig. 1 originally incorrectly read ‘**b–e** HEK293T cells co-expressing FK506-binding protein (FKBP)-NFAST and FKBP-rapamycin-binding domain of mammalian target of rapamycin (FRB)-CFAST*n* (*n* = 10 or 11) were labeled with 5 μM HMBR (4-hydroxy-3-methylbenzylidene rhodanine) (**b**) or 10 μM HBR-3,5DOM (4-hydroxy-3,5-dimethoxybenzylidene rhodanine) (**c**) and imaged before and after the addition of 100 nM rapamycin’. The correct version reads ‘(FKBP)-CFAST*n* (*n* = 10 or 11)’ in place of ‘(FKBP)-NFAST’ and ‘(FRB)-NFAST’ instead of ‘(FRB)-CFAST*n* (*n* = 10 or 11)’.

The sixth sentence of the caption to Fig. 1 originally incorrectly read ‘**e** Temporal evolution of the fluorescence intensity after rapamycin addition in HMBR-treated cells co-expressing FKBP-NFAST and FRB-CFAST11 (*n* = 11 cells; see also Supplementary Figs. 4a, 5a)’. The correct version reads ‘FRB-NFAST’ in place of ‘FKBP-NFAST’ and ‘FKBP-CFAST11’ instead of ‘FRB-CFAST11’.

This has been corrected in both the PDF and HTML versions of the Article.

In Supplementary Figs. 4a and 5a, the labels ‘FKBP’ and ‘FRB’ in the schematic at the top were incorrectly switched, both before and after the reaction.

The second sentence of the caption to Supplementary Fig. 4 originally incorrectly read ‘**(a)** Selected frames of representative HEK293 cells co-expressing FKBP-NFAST and FRB-CFAST*n* (*n* = 10 or 11) (labeled with 5 µM HMBR) upon addition of 100 nM rapamycin’. The correct version reads ‘FRB-NFAST’ in place of ‘FKBP-NFAST’ and ‘FKBP-CFAST*n* (*n* = 10 or 11)’ instead of ‘FRB-CFAST*n* (*n* = 10 or 11)’.

The second sentence of the caption to Supplementary Fig. 5 originally incorrectly read ‘**(a)** Selected frames of representative HEK293 cells co-expressing FKBP-NFAST and FRB-CFAST11 (labeled with 10 µM HBR-3,5DOM) upon addition of 100 nM rapamycin’. The correct version reads ‘FRB-NFAST’ in place of ‘FKBP-NFAST’ and ‘FKBP-CFAST11’ instead of ‘FRB-CFAST11’.

The HTML has been updated to include a corrected version of the Supplementary Information.

