# Supplementary material for: A split fluorescent reporter with rapid and reversible complementation
Source: Nat Commun. 2019 Jun 27;10:2822. doi: 10.1038/s41467-019-10855-0 (PMC6597557; doi:10.1038/s41467-019-10855-0)
Supplement: Supplementary file 1 — Supplementary Information [file 41467_2019_10855_MOESM1_ESM.pdf]

Supplementary Information

A split fluorescent reporter with rapid and reversible complementation

Alison G. Tebo and Arnaud Gautier

Supplementary Table 1. Affinities of the split fragments in presence of FAST's fluorogens.

**CFAST*n***	**Sequence**	**Dissociation constant K_D (μM) of NFAST-CFAST*n*** (in presence of 10 μM HMBR)	**Dissociation constant K_D (μM) of NFAST-CFAST*n*** (in presence of 10 μM HBR-3,5-DOM)
CFAST11	GDSYWVFVKRV	0.21 ± 0.05	1.4 ± 0.2
CFAST10	GDSYWVFVKR	0.95 ± 0.08	6.2 ± 0.5
CFAST9	GDSYWVFVK	5.7 ± 0.6	25 ± 1
CFAST8	GDSYWVFV	21 ± 4	ND

Supplementary Table 2. List of oligonucleotides used in study

Primer code	Sequence
ag126	gtggtgctcgagctattaggaaagggctttcttcatgtgc
ag176	agagtcgcggccgcctattaggaaagggctttcttcatgtgcac
ag175	gcagcggcggagggggatccatggagcatgttgcctttggc
ag181	ggactcagatctgccaccatggaacaaaagcttatttctgaagaggacttggaattcgagatgtggcatgaaggcctg
ag182	ggatccccctccgccgctgccgcctcctccggagacctgctttgagattcgtcgg
ag183	ggactcagatctgccaccatggaacaaaagcttatttctgaagaggacttggaattcggagtgcaggtggaaaccatc
ag184	ggatccccctccgccgctgccgcctcctccggattcttccagttttagaagctccacatc
ag216	ttcgtagctagcatggagcatgttgcctttg
ag311	aaagcttatttctgaagaggacttgtaataggcggccgcgactctagatcataatc
ag313	ctcaccttgctcctgccgagaaagtatcca
ag314	tggatactttctcggcaggagcaaggtgag
ag345	ctagagtcgcggccgcctattaccgtttcacaaagacccaatagc
ag346	ctagagtcgcggccgcctattatttcacaaagacccaatagctgtcac
ag347	taataggcggccgcgactctag
ag358	ggtggcagatctgagtccggtag
ag412	caagtcctcttcagaaataagcttttgttc
ag414	gcttatttctgaagaggacttggtgagcaagggcgaggag
ag415	gaattcgaagcttgagctcgagatctgagtccggacttgtacagctcgtccatgc
ag416	ctcgagctcaagcttcgaattctg
ag417	ccgctgccgcctcctccggaagatctgtatcctggctggaatctag
ag418	gcagcggcggagggggatccatgcccaagaagaagccgac
ag419	caagtcctcttcagaaataagcttttgttcgacgccagcagcatgg
ag455	gactgcgtgacctgtcttattccacttacgacgtgatgagtcgaccatgaattccaagtcctcttcagaaataagc
ag456	ggaataagacaggtcacgcagtcagagctataggtcggctgagctcatccggaggaggcg
ag465	caagtcctcttcagaaataagcttttgttcggatcccttcgctgtcatc

ag466	ggaggaggcggcagcggcggagggggatccgaccaattgactgaagagcagatcgcag
ag467	gctgccgcctcctccggaccgtttcacaaagacccaatag
ag468	caagtccaagggcaaggactccgccgaacaaaagcttatttctgaagaggacttg
ag469	ggagtccttgcccttggacttgatgcagcccatggtggcagatctgagtcc
ag474	ctaccggactcagatctgccaccagtggtgacagctattgggtctttg
ag475	ctagagtcgcggccgcctattacataattacacactttgtctttgacttctttttc
ag535	atccaaaaaagaagagaaaggtagatccaaaaaagaagagaaaggtagatccaaaaaagaagagaaaggtaggta ccgcctccggcgatgaggtggatgg
ag539	ccttgaaattagcaggtcttgatatcgggagctaataggcggccgcgactctag
ag541	ctagagtcgcggccgcctattacacccgtttcacaaagaccc
ag542	ccggactcagatctgccaccatgggtcgtgcgcagtc
ag543	ctacctttctcttctttttggatcggaaagggctttcttcatgtgc
ag544	ccggactcagatctgccaccatgaaggcggagaggaagc
ag545	ctccggcgatgaggtggatggagtgagcaagggcgaggag
ag546	gatatcaagacctgctaatttcaaggctaaggatcccttgtacagctcgtccatgcc
ag550	tgctgaagcaggctggagacgtggaggagaaccctggacctgtgagcaagggcgaggagg
ag694	taactcgaggactacaaggacgacg
ag695	ctcctcgcccttgctcaccatgaattcagcgtaatctggaacatcgtatg
ag696	tggacgagctgtacaagtaataggcggccgcgactc
ag697	cgtcgtccttgtagtcctcgagttaggaaagggctttcttcatgtgcac
ag698	cgtcgtccttgtagtcctcgagttacataattacacactttgtctttgacttctttttc
ag700	cgtcgtccttgtagtcctcgagttaccgtttcacaaagacccaatagc
ag701	gataatatggccacaaccatgcgatcggtgagcaagggcgaggag

Supplementary Table 3. List of plasmids used in study

Plasmid name	Open Reading Frame	Extended Description
pAG144	CFAST11-FRB	CMV-CFAST11-linker-FRB
pAG148	FRB-NFAST	CMV-cmyc-FRB-linker-NFAST
pAG149	FKBP-NFAST	CMV-cmyc-FKBP-linker-NFAST
pAG152	FRB-CFAST11	CMV-cmyc-FRB-linker-CFAST11
pAG153	FKBP-CFAST11	CMV-cmyc-FKBP-linker-CFAST11
pAG209	Histag-NFAST	pET28a-Histag-NFAST
pAG241	FKBP-CFAST10	CMV-cmyc-FKBP-linker-CFAST10
pAG296	mCherry-ERK2-CFAST10	CMV-myc-mCherry-ERK-CFAST10
pAG298	NFAST-MEK1	CMV-NFAST-MEK-myc
pAG301	NFAST-MKP1	CMV-NFAST-MKP1-myc
pAG334	M13-NFAST	CMV-myc-M13-NFAST
pAG335	CFAST10-CaM	CMV-CFAST10-CaM-myc
pAG336	lyn11-FRB-NFAST	CMV-lyn11-cmyc-FRB-NFAST
pAG340	CFAST10-Raf1-mCherry	CMV-CFAST10-Raf1-mCherry-myc
pAG341	NFAST-KRas	CMV-NFAST-myc-KRas
pAG384	bFos-CFAST11	CMV-bFos-myc-CFAST11
pAG385	bJun-NFAST-NLS-DEVDG-mCherry-NES	CMV-bJun-myc-NFAST-NLSx3-DEVDG-mCherry-NES
pAG490	FRB-NFAST-IRES-mTurquoise2	CMV-cmyc-FRB-NFAST-IRES-HA-mTurquoise2
pAG491	lyn11-FRB-NFAST-IRES-mTurquoise2	CMV-lyn11-cmyc-FRB-NFAST-IRES-HA-mTurquoise2
pAG492	NFAST-MEK1-IRES-mTurquoise2	CMV-NFAST-MEK-myc-IRES-HA-mTurquoise2

pAG493	NFAST-MKP1-IRES-mTurquoise2	CMV-NFAST-MKP1-myc-IRES-HA-mTurquoise2
pAG494	NFAST-KRas-IRES-mTurquoise2	CMV-NFAST-myc-KRas-IRES-HA-mTurquoise2
pAG496	FKBP-CFAST10-IRES-mCherry	CMV-myc-FKBP-CFAST10-IRES-mCherry-myc
pAG439	FRB-NFAST-P2A-mCherry	CMV-myc-FRB-NFAST-P2A-mCherry

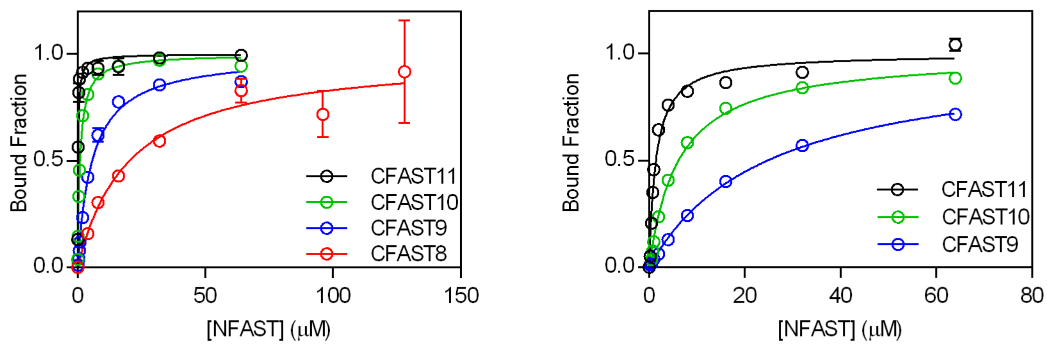

Supplementary Figure 1. In vitro binding affinities of NFAST and CFAST*n* (*n* = 11, 10, 9 or 8) in the presence of 10 μM HMBR (left) or 10 μM HBR-3,5DOM (right). Titrations were done at a CFAST*n* concentration of 100 nM in pH 7.4 phosphate buffered saline at 25 °C. Excitation was fixed at 480 nm and emission at 540 nm for HMBR and 520 nm and 600 nm for HBR-3,5DOM. Data represent mean ± sem (n = 3). Least square fit (line) gave the thermodynamic dissociation constants K_D presented in **Supplementary Table 1**.

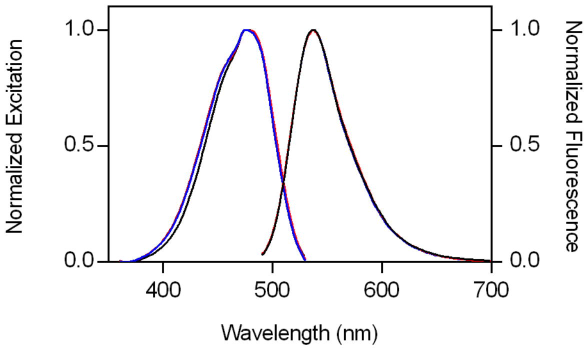

Supplementary Figure 2. Excitation and emission spectra of FAST:HMBR (blue), splitFAST11:HMBR (red), and splitFAST10:HMBR (black). splitFAST11 (resp. splitFAST10) results from the complementation of NFAST and CFAST11 (resp. CFAST10).

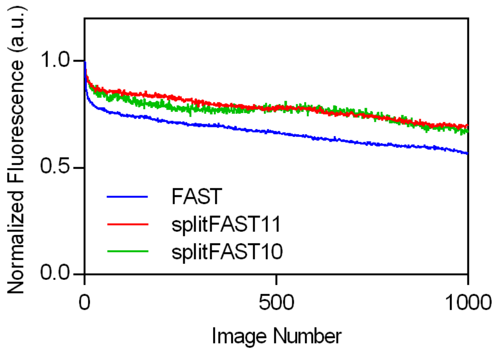

Supplementary Figure 3. Evolution of the cellular fluorescence of HMBR-labeled HEK293 cells expressing FAST, splitFAST11, and splitFAST10 upon imaging by confocal microscopy. Cells expressing FRB-NFAST and FKBP-CFAST*n* (*n* = 10 or 11) were treated with rapamycin to form splitFAST11 and splitFAST10. Cells expressing FAST were used as control. HMBR concentration was 10 µM. Excitation at 488 nm at 6.3 kW/cm^2. Six cells analyzed per condition.

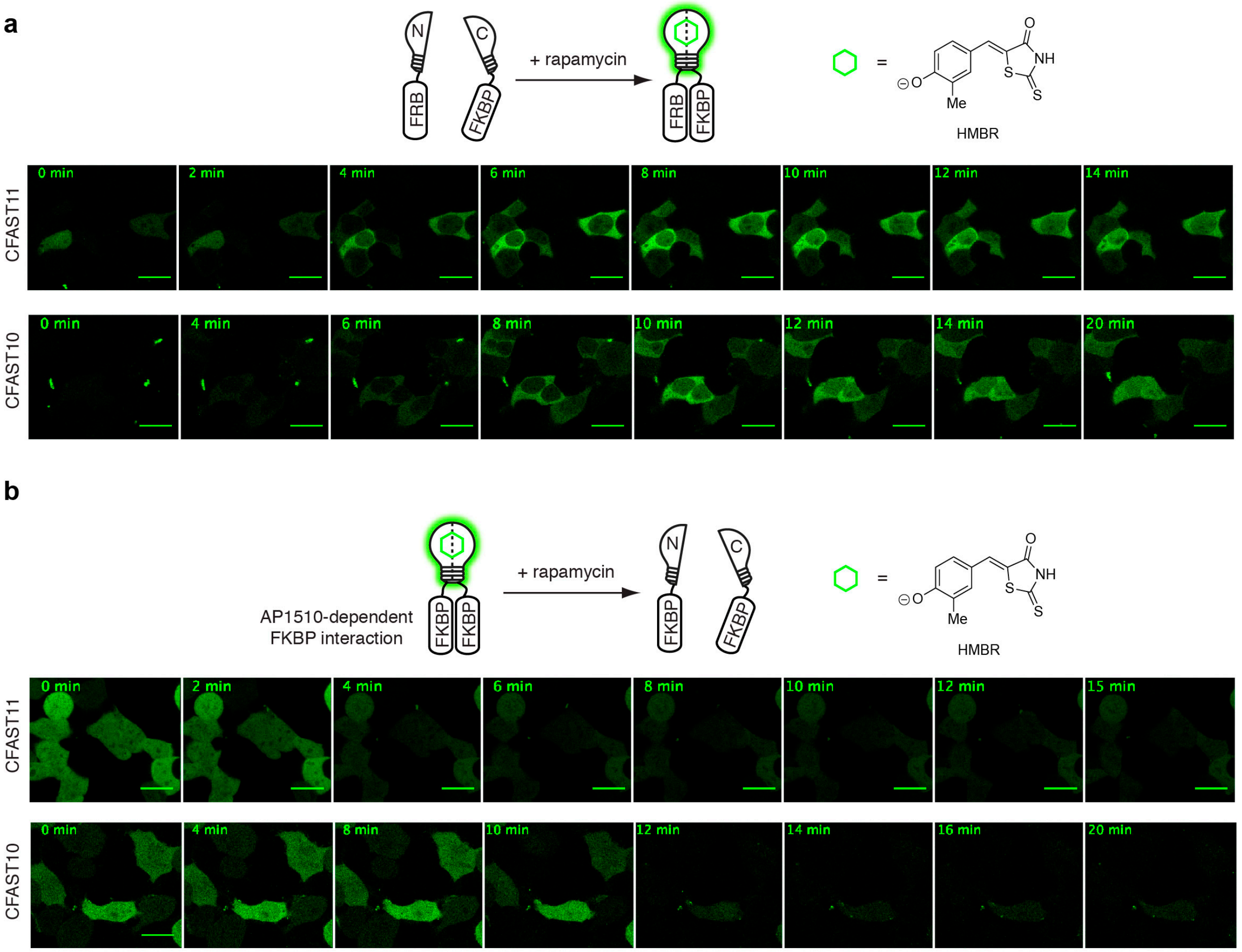

Supplementary Figure 4. Rapid and reversible complementation of splitFAST:HMBR. (a) Selected frames of representative HEK293 cells co-expressing FRB-NFAST and FKBP-CFAST*n* (*n* = 10 or 11) (labeled with 5 μM HMBR) upon addition of 100 nM rapamycin. **(b)** Selected frames of representative AP1510-treated HEK293 cells co-expressing FKBP-NFAST and FKBP-CFAST*n* (*n* = 10 or 11) (labeled with 5 μM HMBR) upon addition of 1 μM rapamycin. Scale bars 20 μm.

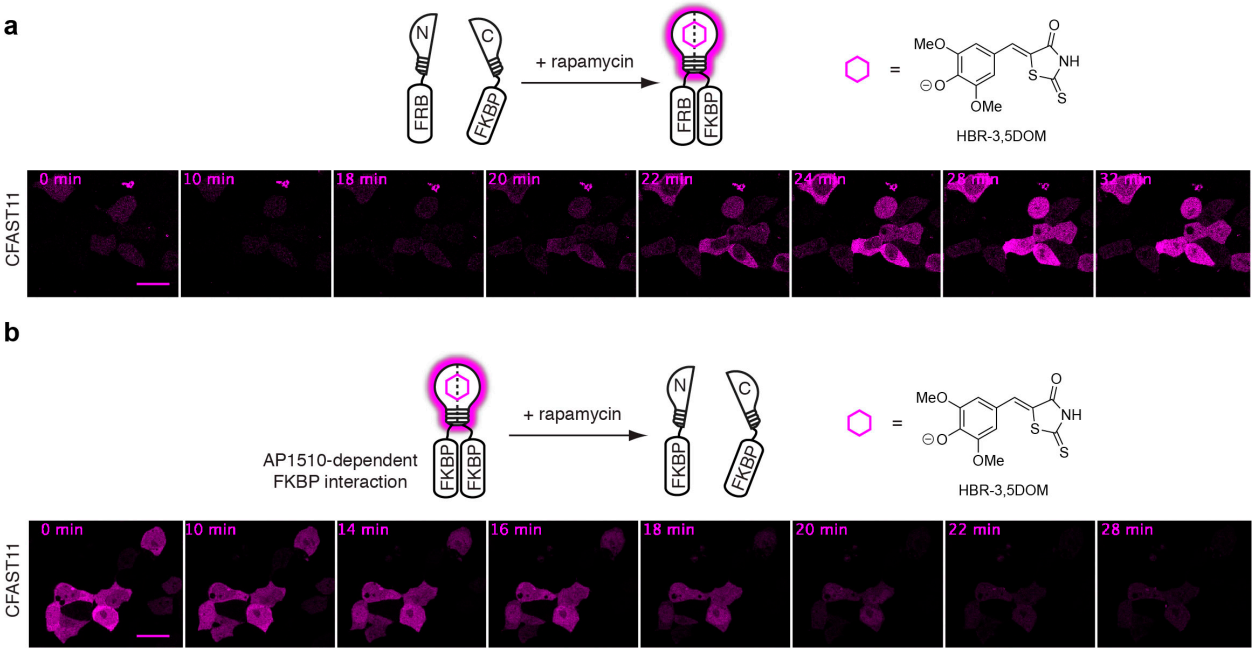

Supplementary Figure 5. Rapid and reversible complementation of splitFAST:HBR-3,5DOM. (a) Selected frames of representative HEK293 cells co-expressing FRB-NFAST and FKBP-CFAST11 (labeled with 10 μM HBR-3,5DOM) upon addition of 100 nM rapamycin. **(b)** Selected frames of representative AP1510-treated HEK293 cells co-expressing FKBP-NFAST and FKBP-CFAST11 (labeled with 10 μM HBR-3,5DOM) upon addition of 1 μM rapamycin. Scale bars 20 μm.

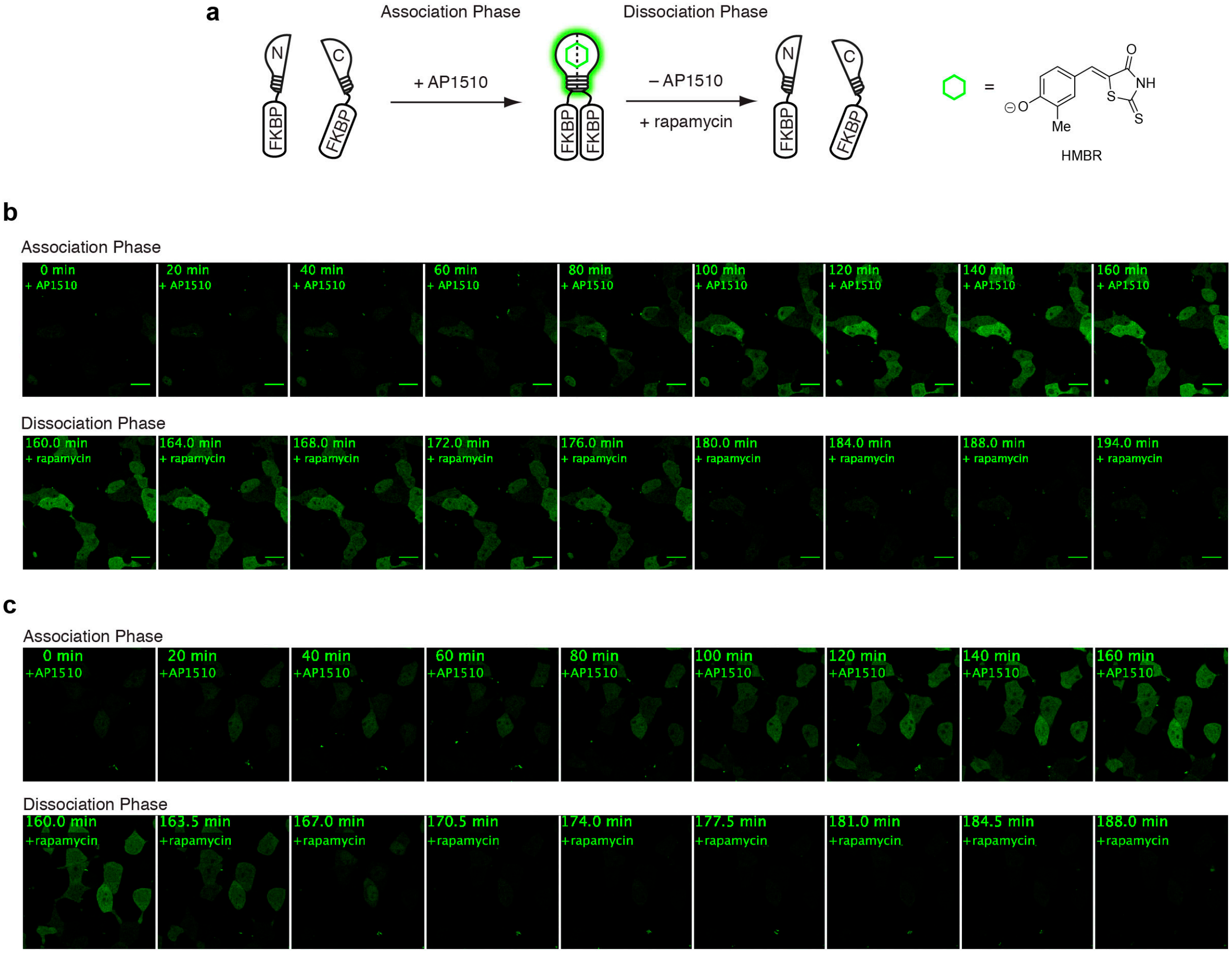

Supplementary Figure 6. Rapid and reversible complementation of splitFAST in a single experiment. (a-c) HMBR-labeled cells co-expressing FKBP-NFAST and FKBP-CFAST*n* (*n* = 11 **(b)** or 10 **(c)**) were firstly treated with 100 nM AP1510 for 160 min (Association Phase), then AP1510 was removed, and 1 μM rapamycin was added (Dissociation Phase). Selected frames are shown (see also **Supplementary Movies 1** and **2**). Scale bars 30 μm.

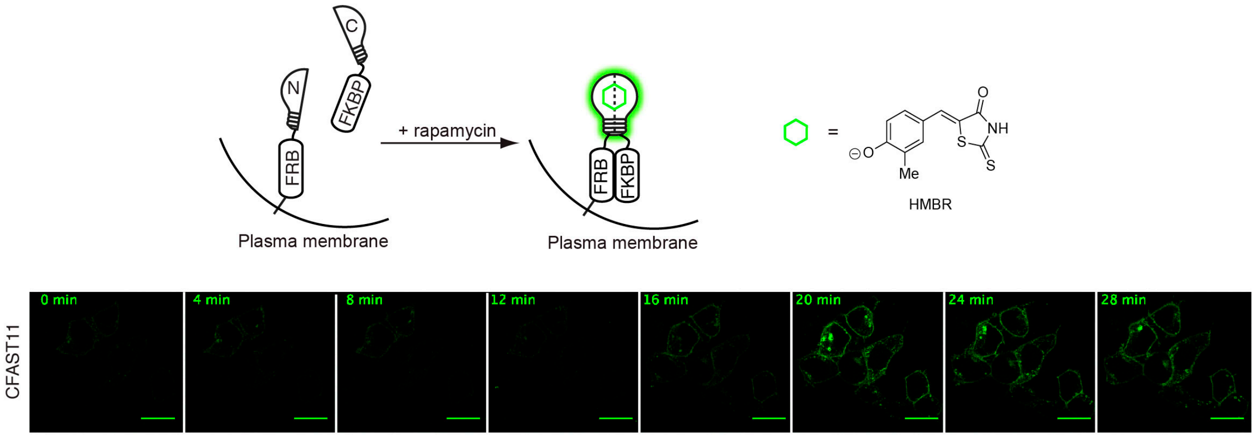

Supplementary Figure 7. Rapid complementation of splitFAST at the plasma membrane. Selected frames of representative HEK293 cells co-expressing Lyn11-FRB-NFAST and FKBP-CFAST11 (labeled with 5 μM HMBR) upon addition of 100 nM rapamycin. Scale bars 20 μm.

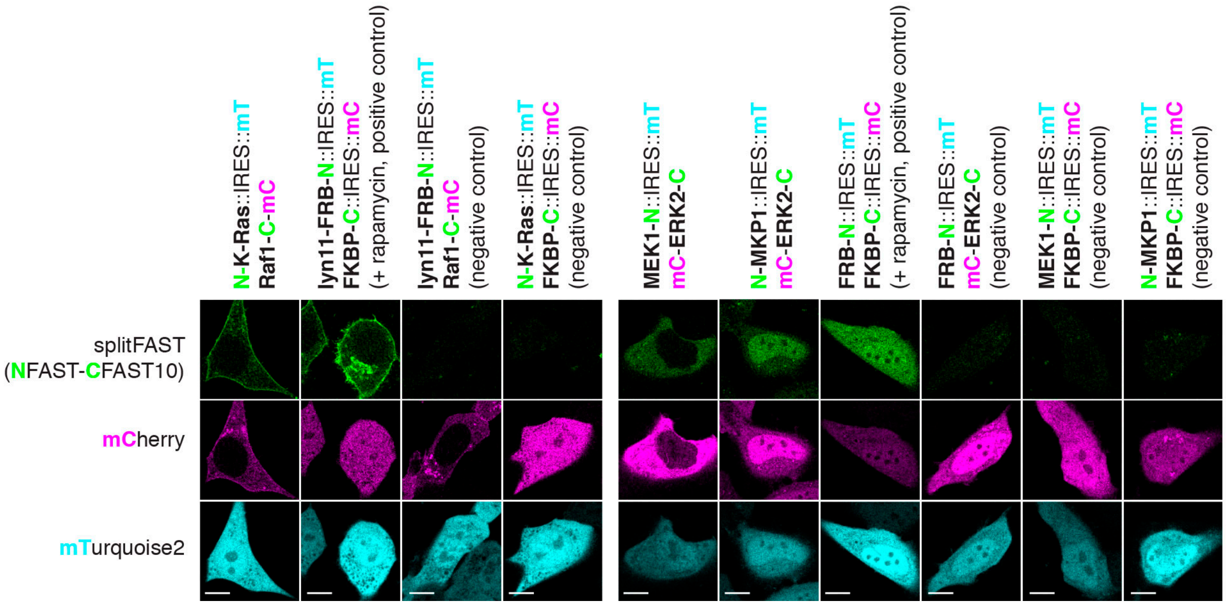

Supplementary Figure 8. Use of splitFAST for imaging K-Ras/Raf1, MEK1/ERK2 and ERK2/MKP1 interactions. Representative cells co-expressing the indicated constructs were imaged in presence of 10 μM HMBR. Positive and negative controls are shown. Scale bars 10 μm.
