# Supplementary material for: A split fluorescent reporter with rapid and reversible complementation
Source: Nat Commun. 2019 Jun 27;10:2822. doi: 10.1038/s41467-019-10855-0 (PMC6597557; doi:10.1038/s41467-019-10855-0)
Supplement: Supplementary file 3 — Description of Additional Supplementary Files [file 41467_2019_10855_MOESM3_ESM.pdf]

Description of Additional Supplementary Files

File name: Supplementary Movie 1

Description: Use of splitFAST for imaging the association and dissociation of FKBP homodimer. Time-lapse imaging of HMBR-labeled HEK293 cells co-expressing FKBP-NFAST and FKBP-CFAST11 upon, firstly, treatment with 100 nM AP1510, and, secondly, removal of AP1510 at t = 160 min and addition of 1 μM rapamycin (see also **Figure 1j,k** and **Supplementary Figure 6b**). Scale bar 30 μm.

File name: Supplementary Movie 2

Description: Use of splitFAST for imaging the association and dissociation of FKBP homodimer. Time-lapse imaging of HMBR-labeled HEK293 cells co-expressing FKBP-NFAST and FKBP-CFAST10 upon, firstly, treatment with 100 nM AP1510, and, secondly, removal of AP1510 at t = 160 min and addition of 1 μM rapamycin (see also **Figure 1j** and **Supplementary Figure 6c**). Scale bar 30 μm.

File name: Supplementary Movie 3

Description: Use of splitFAST for imaging the evolution of MEK1/ERK2 interaction upon EGF stimulation. Time-lapse imaging of a representative HMBR-labeled Hela cell co-expressing MEK1-NFAST and mCherry-ERK2-CFAST10 after stimulation with EGF. splitFAST channel is shown on the left-hand side, while mCherry channel is shown on the right-hand side (see also **Figure 2b-d**).

File name: Supplementary Movie 4

Description: Use of splitFAST for imaging of the Ca2+-dependent interaction of Calmodulin (CaM) and the Ca2+-CaM interacting peptide M13. The sensor is composed of M13-NFAST and CFAST10-CaM. Time-lapse imaging of a representative HeLa cell treated with histamine (see also **Figure 2e-g**). Scale bar 20 μm.

File name: Supplementary Movie 5

Description: Use of splitFAST for detecting caspase-3 activity. Two-color time-lapse imaging of representative HMBR-labeled cells expressing bFos-CFAST11 and bJun-NFAST-NLS3-DEVDG-mCherry-NES after treatment with staurosporine (see also **Figure 2h-j**). The mCherry signal is in magenta while the splitFAST signal is in green. Scale bar 20 μm.
